# Persistent task-specific impairment of holistic face processing in acquired prosopagnosia

**DOI:** 10.1038/s41598-025-28666-3

**Published:** 2025-12-04

**Authors:** Bryan Qi Zheng Leong, Ahamed Miflah Hussain Ismail, Alejandro J. Estudillo

**Affiliations:** 1 School of Psychology, Bournemouth University, Poole House Talbot Campus, BH12 5BB Bournemouth, UK; 2 School of Psychology, University of Nottingham Malaysia, Semenyih, 43500 Selangor Malaysia

**Keywords:** Acquired prosopagnosia, Face recognition, Holistic processing, Inversion effect, Composite effect, Part-whole effect, Single-case analysis, Cognitive neuroscience, Learning and memory, Regeneration and repair in the nervous system, Sensory processing

## Abstract

Face recognition deficits observed in Acquired Prosopagnosia are typically associated with impaired holistic processing. Nonetheless, whether this deficit is task-dependent, face-specific, and persistent over time has been under investigated. The present study examined the role of holistic processing in a case of acquired prosopagnosia (Patient DS). Patient DS, along with several neurotypical participants, completed the three standard measures of holistic face processing: the face inversion, part-whole, and composite face tasks, as well as a measure of non-face global processing: the Navon task. Our single-case analyses indicated that, compared to neurotypical participants, DS showed (1) impaired inversion effects, but (2) comparable part-whole and composite face effects, as well as (3) comparable global precedence effect in the Navon task. Interestingly, the same pattern of preserved and impaired holistic processing was observed in a second evaluation of DS four years later. While these findings suggest that holistic processing deficits in Acquired Prosopagnosia may be *task-specific* and *persist* over time—pointing toward the potential chronicity of the impairment—they should be interpreted with caution, given the floor effects and the single-case design.

**Supplementary Information:**

The online version contains supplementary material available at 10.1038/s41598-025-28666-3.

## Introduction

The human face recognition system is typically linked with the occipito-temporal cortex, particularly in the right hemisphere^1,2^. Lesions in these regions often result in Acquired Prosopagnosia (AP), a neuropsychological condition marked by a profound deficit in face recognition while sparing the recognition of non-face objects^3–6^. Individuals with AP typically exhibit difficulties in recognising previously familiar faces or forming new face memories following brain injury, despite having intact low-level visual processing and normal intelligence (see review by Benton^7^. Early accounts of AP^8^^[,9^ suggested that these individuals show a general impairment in integrating individual elements into a unified percept (i.e., holistic processing), irrespective of whether the stimuli are faces or non-face objects (i.e., Navon letters^10^. Additional support for this view comes from Barton et al.^11^, who found that several AP cases had difficulty recognising overlapping figures or reconstructing fragmented letters. Nonetheless, more recent accounts have emphasized on a face-specific impairment in holistic processing^5^.

While coarsely defined, holistic processing remains a central concept in the face processing literature^12–17^. According to the holistic account, face recognition relies on integrating facial features into a perceptual whole, rather than isolated processing of individual facial features^13,18–20^. In typical adults, holistic processing in face recognition is predominantly demonstrated using three gold-standard paradigms: the *face inversion*, the *part-whole*,* and* the *composite face* tasks (for detailed description, see Leong et al.^14^; Rezlescu et al.^15^). In the inversion task^21,22^, recognition is impaired for *inverted* faces (control condition), compared to *upright* faces (experimental condition), since the former disrupts holistic processing. In the composite face task^16,23,24^, an illusory identity is perceived when one half of an identity’s face is spatially *aligned* with another half of a different identity (experimental condition). However, when the two halves are *misaligned* (control condition), holistic processing is disrupted, and this illusion disappears. In the part-whole task^14,25–27^, identification tends to be more accurate for a previously learned face when it is shown within the context of a *whole* face (experimental condition), as opposed to an isolated *part* (control condition). This is because facial features are thought to be encoded through integrating individual parts into a unified whole.

The holistic processing account of AP is supported by studies that have found abolished or atypical effects in the inversion task^28^, the part-whole task^29^, the composite face task^30^, or sometimes, in all three of those tasks^5,31–33^. This was the case for Patient PS^34^^[,35^, who showed comparable performance to demographically-matched neurotypical controls (NTs) in the Navon task but had severe impairments in holistic face processing, as evidenced by deficits across all three gold-standard paradigms of holistic processing^32,33^. However, this account has been challenged by more recent findings, with one study reporting that five out of seven APs demonstrated normal composite face effects for upright faces^36^ (see also Rezlescu et al.^6^ for similar findings). The authors^36^ proposed that holistic processing may remain intact in some APs because holistic face information is represented across multiple “regions” within the face processing network (for discussion, see also Duchaine & Yovel^37^. Thus, some APs are still efficient in processing degraded visual inputs relayed from damaged regions^36^.

Notably, a major weakness of these previous studies is the assumption that the three gold-standard paradigms of holistic processing tap into the same underlying cognitive mechanism(s). However, more recent evidence suggest that these three holistic face measures indeed reflect different underlying cognitive mechanisms^12,14,15,38,39^. In a recent study, Rezlescu et al.^15^ found that performances in these three measures were only weakly or not associated with each other (e.g., the composite face effect was not correlated to either the face inversion or the part-whole effects). These findings suggest that holistic processing is a multifaceted construct and that a common mechanism may not underlie the three putative effects observed in holistic processing tasks (see also review by Boutet et al.^12^). Furthermore, Rezlescu et al.^15^ also found varying associations between the inversion and the part-whole effects with face identification, as measured by the Cambridge Face Perception Test (CFPT^40^. In contrast, the composite effect was not associated with face identification^15^. This suggests that individual differences seen in holistic face recognition are task-dependent. More recently, in our lab, we revealed that developmental prosopagnosics (DPs) showed impairments in the inversion and part-whole effects, but not in the composite effect, compared to neurotypical controls^14^. Additionally, using single-case analyses, our findings revealed that DPs exhibit distinct holistic deficits (e.g., some DPs were impaired only in the inversion task, while others were impaired only in the part-whole task), supporting the so-called *heterogeneous* holistic processing deficit hypothesis^14^. Overall, these findings provide converging evidence that the underlying construct of holistic processing is not unitary.

In light of this, heterogeneity of impairments in DPs may affect different aspects of holistic face processing. Thus, it is possible that, like DPs, APs may also exhibit impairments that are specific to distinct cognitive mechanism(s) as measured by the three holistic processing paradigms. This hypothesis needs to be further tested, as the previously reviewed patient studies neither confirm nor rule out these possibilities. For example, the fact that Patient PS showed deficits across the three holistic processing paradigms, does not mean that these tasks reflect the same cognitive mechanisms, since an association between tasks do not rule out a dissociation at a cognitive level^41,42^. Additionally, although some of the APs in Finzi et al.’s^36^ study, did not show deficits in holistic processing as measured by the composite face task, it is still possible that holistic deficits would have been revealed if measured using the face inversion or the part whole tasks.

Nevertheless, fundamental differences in etiology and compensatory potential limit the extent to which DP findings can inform AP research^43^. Unlike DP, AP involves direct neural damage, which makes their deficit vary widely across individuals, and the deficits are not always selective to face processing^44–46^. Further, DPs often develop compensatory strategies over their lifetime to cope with their face deficits^47^, which are rarer in APs due to the sudden onset and more widespread damage to their face neural structure^48^. Moreover, while compensatory and remedial trainings have shown some improvements in DPs, similar interventions have yielded more limited or short-lived gains in APs^48–51^. These differences between DPs and APs further highlights the importance of assessing the temporal stability and mechanism-specific deficits in AP, particularly in relation to holistic face processing.

### The present study

Here, we present Patient DS, an Acquired Prosopagnosic with face recognition difficulties following an ischemic stroke. Patient DS completed the three gold-standard paradigms of holistic face processing. In addition, to examine whether holistic processing deficits of Patient DS (if any) are specific to faces, we also measured their ability to holistically process non-face objects using the Navon’s task (i.e., alphabetical characters). The same tasks were also completed by a group of neurotypical participants, who served as our comparison to Patient DS.

A novel and key aspect of this study is the investigation of the temporal stability of holistic processing for both faces and non-face objects in AP. Longitudinal studies on Acquired Prosopagnosia are scarce, and importantly, it is unclear whether holistic face processing can be improved, relearned, or even deteriorate over time in AP. For instance, APs might learn or develop compensatory strategies to recognize faces over time, potentially resulting in normal performance on these tasks at one time point but not another, suggesting that holistic processing can be rehabilitated. Conversely, if the underlying mechanism(s) of holistic processing are linked to distinct brain regions, we would expect that damage to these regions are irreversible and therefore, we can observe task-specific impairments to persist over time. To explore whether holistic processing skills in Acquired Prosopagnosia are stable over time, we tested Patient DS for a second time using the same holistic processing measures, four years after the initial evaluation.

### Data analysis

First, we wanted to determine if Patient DS was in fact a case of “pure” acquired prosopagnosia^5,31,33,52,53^. To do this, we conducted two different modified *t*-tests designed for single-case analyses. These statistical approaches address the challenge of variability within an Acquired Prosopagnosia individual, while allowing for comparison against a control group^14^, making it an appropriate choice for the current study. The first test (i.e., Singlims_ES.exe^54,55^ was conducted to compare performances between DS and the demographically-matched NT group (*N* = 15) in the evaluation stage, which consisted of objective measures of face recognition (i.e., Cambridge Face Memory Test; CFMT^56^, face perception (CFPT^40^, and non-face object recognition (i.e., Cambridge Car Memory Test; CCMT^57^.

While Crawford and his colleagues^54,55^ may have suggested the appropriateness of the modified *t*-test when dealing with small control samples, a larger control sample would produce a more reliable normative range. Therefore, we also compared DS against a larger group of NTs that were not age-matched (*N* = 45). To account for the potential influence of age^58,59^, we used Crawford et al.’s single-case Bayesian analysis (i.e., BTD_Cov.exe^60^, including age as a covariate. This approach enabled us to determine whether any observed differences between DS and age-matched NTs persisted even after statistically controlling for age and comparing DS against a larger NT sample.

In the second test, we wanted to confirm that our different holistic face measures were consistent with findings from other studies. For this, we ran multiple repeated-measures *t*-tests to compare the performances of NTs in the *condition of interest* (i.e., upright, whole, and same-aligned trials) with the corresponding *control conditions* (i.e., inverted, part, same-misaligned trials) in the face inversion, part-whole, and composite face tasks, respectively.

Holistic processing (i.e., the holistic advantage) in these tasks has been traditionally indexed using subtraction methods^13,15,61^. However, this method does not account for the variation in performances that the condition of interest shares with the control condition (for a discussion, see DeGutis et al.^61^; Leong et al.^13^). For this reason, the “regression” method was used to index holistic advantage in the present study. Accordingly, to examine group-level differences in holistic processing for each holistic measure, we used the control-based regression approach^14,62^ in which the variances of the *control conditions* are regressed from the variances in the *condition of interest*, for the data from the NT group. We included only NTs’ data to ensure that the regression models were based on normative performance. These models were then used to calculate the residuals for Patient DS (for calculations, see Leong et al. ^14^; Berger et al.^63^).

To index holistic advantage in the Navon’s task (i.e., global precedence), we calculated the standardised mean difference (Cohen’s *d*) between response times (RTs) for accurate responses provided by the participants in the Local congruent and Global congruent trials^14,64^. This index is not confounded with interference effects^64,65^, thus reflecting a purer global precedence index compared to other Navon indices. These standardised differences were then compared between Patient DS and NTs. Accordingly, we also ran modified *t*-tests^54,55,60^ comparing the residual scores (e.g., inversion, part-whole and composite effect) and standardised differences (Navon’s task), between Patient DS and NTs.

Finally, to test the temporal stability of holistic deficits in Patient DS, we applied another modified *t*-test that incorporates classical inferential approaches for comparing the scores of two single-cases while referring the cases’ scores to a control sample (i.e., C_CTC.exe; Classical method Compare Two Cases)^66^. Conventionally, studies have used this test to examine whether the differences in performances of two independent single cases are significantly greater than the variability in differences observed within the control sample^66–69^. Using a similar logic, an adaptation of this approach was used to assess changes in performance across two time points in the same individual. In other words, Patient DS’ first and second evaluations were treated as two “cases”. The test then statistically compares the differences between the first and second evaluations against the distribution of differences in age-matched NTs (*N* = 15). This approach has previously been used to compare the performance of neuropsychological patients at two different time points^70,71^. Further, to assess the temporal stability of holistic deficits in the presence of a covariate (i.e., age), we also compared DS’ first and second evaluations against the distribution of differences in the larger sample of NTs (*N* = 45) using an extended version of the former test that includes age as a covariate (i.e., CTC_Cov.exe)^66^.

## Results

A summary of Patient DS (see the Methods section for patient history) and neurotypical controls’ (NTs) performances are summarized in Table 1. First, our modified *t*-tests indicated impaired performance of Patient DS compared to age matched-NTs in face recognition (CFMT) and face perception (CFPT), but normal non-face object recognition (CCMT), confirming DS as a case of pure Acquired Prosopagnosia (see Table 2). These results were replicated using a larger sample of NTs (see Table 3).

**Table 1:** *The accuracy (CFMT*,* CFPT*,* CCMT*,* inversion*,* part-whole and composite tasks) and reaction time (Navon’s task) performances between patient DS and the respective age matched*-*NTs and larger sample NTs.*

Tasks/Conditions	Patient DS		Age matched-NTs		All NTs	
	M_1_	M_2_	M	SD [± 95% CI]	M	SD [± 95% CI]
**CFMT**	32	-	62.8	9.886 [5.475]	59.1	8.921 [2.680]
**CFPT**	98	-	48.3	8.447 [4.678]	51.6	11.887 [3.571]
**CCMT**	49	-	53.4	10.246 [5.674]	49.9	9.230 [2.773]
**Inversion**						
Upright	0.250	0.450	0.843	0.126 [0.070]	0.799	0.128 [0.038]
Inverted	0.375	0.350	0.588	0.095 [0.053]	0.570	0.140 [0.042]
**Part-whole**						
Whole	0.556	0.528	0.796	0.096 [0.053]	0.779	0.104 [0.031]
Part	0.500	0.472	0.728	0.048 [0.027]	0.694	0.073 [0.022]
**Composite**						
Aligned	0.625	0.475	0.778	0.153 [0.085]	0.738	0.164 [0.049]
Misaligned	0.625	0.550	0.883	0.125 [0.069]	0.828	0.187 [0.056]
**Navon’s (ms)**						
Global	2922.5	770.0	887.5	711.6 [394.1]	848.2	684.8 [205.7]
Local	2837.0	791.5	949.8	711.7 [394.1]	917.7	655.6 [206.0]

Note. *M*_*1*_, first evaluation; *M*_*2*_, second evaluation; ± 95% CI, confidence interval values above and below the mean.

**Table 2:** Single-case analyses comparing patient DS against age-matched NTs.

Tasks		Patient DS	
		*M*_1_	*M*_2_
CFMT	Sum	−30.8	-
	*t*	−3.016**	-
	*Zcc*	−3.115	-
	95% CI	[−4.354, −1.858]	-
CFPT	Error mean	50.3	-
	*t*	5.701***	-
	*Zcc*	5.888	-
	95% CI	[3.669, 8.069]	-
CCMT	Sum	−4.4	-
	*t*	− 0.416	-
	*Zcc*	− 0.429	-
	95% CI	[−0.953, 0.108]	-
FIE	Residuals	− 0.435	− 0.225
	*t*	−3.716**	−2.183*
	*Zcc*	−3.838	−2.255
	95% CI	[−5.324, −2.337]	[−3.213, −1.275]
PWE	Residuals	− 0.100	− 0.110
	*t*	−1.043	−1.162
	*Zcc*	−1.077	−1.200
	95% CI	[−1.707, − 0.423]	[−1.859, − 0.517]
CFE	Residuals	− 0.002	− 0.117
	*t*	− 0.257	−1.204
	*Zcc*	− 0.265	−1.243
	95% CI	[−0.776, 0.255]	[−1.912, − 0.550]
GPE	Cohen’s *d*	− 0.093	0.219
	*t*	−1.224	− 0.419
	*Zcc*	−1.264	− 0.432
	95% CI	[−1.939, − 0.566]	[−0.956, 0.105]

Note. *M*_*1*_, differences in first evaluation between DS and matched-NTs; *M*_*2*_, differences in second evaluation between DS and matched-NTs; Residual scores are based on the normative regression lines of matched-NTs’ performances, wherein a significantly different score between Patient DS and the demographically-matched NTs are highlighted in grey; *Z*cc, Effect size for difference between case and controls; 95% CI, 95% confidence interval estimates of the effect size; modified independent *t*-test: *p <. 05, **p <.01, ***p <.001 (two-tailed).

**Table 3:** Single-case analyses comparing patient DS against a larger group of NTs (*N* = 45) across both timepoints, after controlling for age.

Tasks		Patient DS	
		*M*_1_	*M*_2_
CFMT	Sum	−27.133	-
	*z*-score	−3.042**	-
	*Zccc*	−3.012	-
	95% CI	[−3.679, −2.265]	-
CFPT	Error mean	46.4	-
	*z*-score	3.902***	-
	*Zccc*	4.151	-
	95% CI	[3.177, 5.023]	-
CCMT	Sum	− 0.911	-
	*t*	− 0.099	-
	*Zccc*	− 0.013	-
	95% CI	[−0.341, 0.315]	-
FIE	Residuals	− 0.490	− 0.282
	*z*-score	−4.256***	−2.453*
	*Zccc*	−4.254	−2.348
	95% CI	[−5.144, −3.258]	[−2.923, −1.706]
PWE	Residuals	− 0.164	− 0.184
	*z*-score	−1.726	−1.930
	*Zccc*	−1.655	−1.810
	95% CI	[−2.105, −1.155]	[−2.306, −1.260]
CFE	Residuals	− 0.043	− 0.167
	*z*-score	− 0.307	−1.203
	*Zccc*	− 0.329	−1.236
	95% CI	[−0.658, 0.011]	[−1.662, − 0.771]
GPE	Cohen’s *d*	− 0.567	− 0.145
	*z*-score	−1.128	− 0.289
	*Zccc*	−1.115	− 0.270
	95% CI	[−1.499, − 0.696]	[−0.633, 0.103]

Note. *M*_*1*_, differences in first evaluation between DS and all NTs; *M*_*2*_, differences in second evaluation between DS and all NTs; Residual scores are based on the normative regression lines of all NTs’ performances (*N* = 45), wherein a significantly different score between Patient DS and the NTs are highlighted in grey; *Z*ccc, Effect size for difference between case and controls; 95% CI, 95% confidence interval estimates of the effect size; modified independent *t*-test: *p <. 05, **p <.01, ***p <.001 (two-tailed).

All repeated-measures *t*-tests revealed statistically significant differences between the condition of interest and control conditions, effectively capturing holistic processing. Specifically, in the face inversion task, face recognition was significantly more accurate in the upright condition than the inverted condition (*t*(14) = 6.146, *p* <.001, *d* = 1.587). In the part-whole task, accuracy was significantly higher in the whole condition compared to the part condition (*t*(14) = 3.150, *p* =.007, *d* = 0.813). Similarly, in the composite face task, recognition of the top parts of faces was more accurate in the same-misaligned condition than the same-aligned condition (*t*(14) = 3.877, *p* =.002, *d* = 1.001). This replicates the past literature to reveal clear face inversion, part-whole, and composite face effects.

**Fig. 1:**
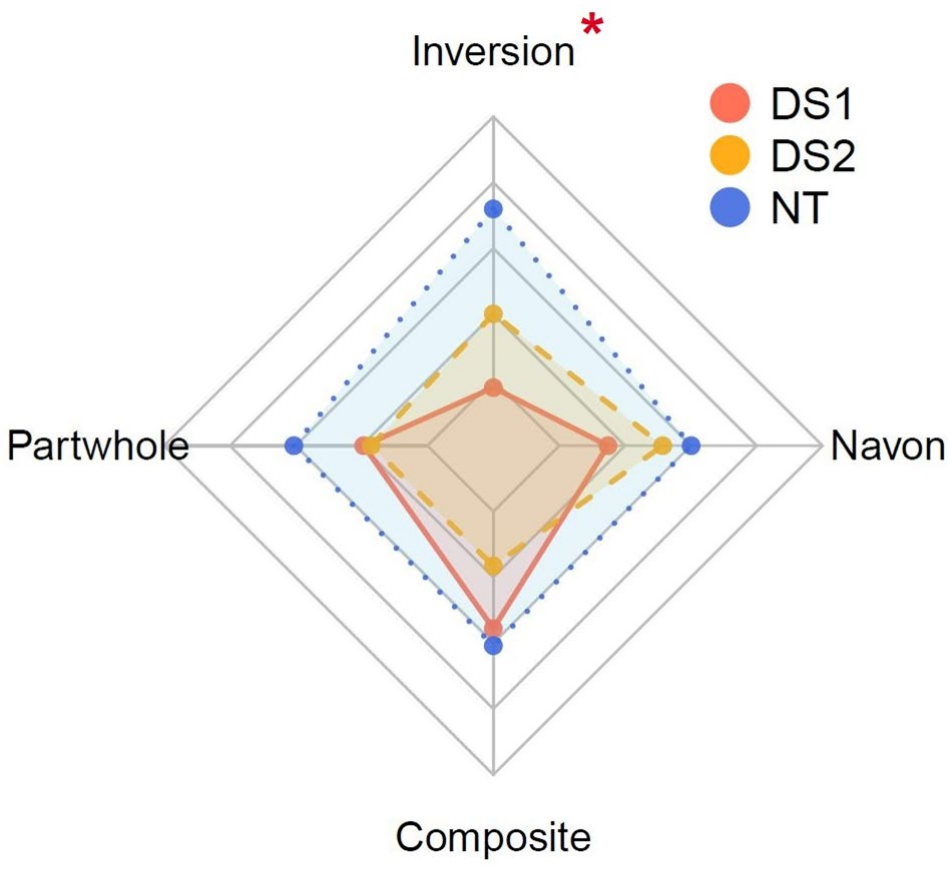
Patient DS (two evaluations) and demographically-matched NTs performances in the four holistic measures. Note. *DS1*, first evaluation of Patient DS; *DS2*, second evaluation of Patient DS; The maximum (outermost grid) and minimum (innermost grid) threshold of each subscale are ± 2 standard deviations from the mean (centre grid) of residual scores (face inversion, part-whole and composite face tasks) and standardized differences (Navon’s task) relative to their demographically-matched NTs (*N* = 15). Each scale contains 4 segments, in which each segment represents ± 1 SD from the mean. A red asterisk (*) signifies that the differences in performance were significant between Patient DS and NTs (two-tailed).

Additionally, our single-case analyses identified that Patient DS was selectively impaired in one out of the four holistic measure(s) when compared to demographically-matched NTs (see Fig. 1). Specifically, Patient DS was impaired in the inversion effect, but not the part-whole, composite, or global precedence effects (in the Navon task), when compared to matched-NTs and also the larger sample of NTs (refer to Tables 2 and 3, respectively). Interestingly, this specific impairment was persistent in the second evaluation four years later in both analyses (see Tables 2 and 3).

Lastly, to test the temporal stability of holistic deficits in Patient DS, we compared DS’ first and second evaluations against the distribution of differences in matched-NTs and the larger sample of NTs. Overall, no significant differences (two-tailed) were found between the two evaluations in the face inversion (*t*(14) = −1.118, *p* =.282, *Z-PCC* 95% *CI* [−1.528, − 0.709]), the part-whole (*t*(14) = 0.079, *p* =.937, *Z-PCC* 95% *CI* [0.050, 0.109]), the composite face (*t*(14) = 0.692, *p* =.500, *Z-PCC* 95% *CI* [0.439, 0.945]), and the global precedence (*t*(14) = − 0.574, *p* =.575, *Z-PCC* 95% *CI* [−0.784, − 0.364]) effects. The extended test with the larger sample of NTs, factoring for age, also revealed no significant differences (two-tailed) between the two evaluations in the face inversion (*t*(43) = −1.333, *p* =.190, *Z-PCCC* 95% *CI* [−1.048, −1.618]), the part-whole (*t*(43) = 0.108, *p* =.914, *Z-PCCC* 95% *CI* [0.161, 0.054]), the composite face (*t*(43) = 0.633, *p* =.530, *Z-PCCC* 95% *CI* [0.775, 0.491]), and the global precedence (*t*(43) = − 0.591, *p* =.558, *Z-PCCC* 95% *CI* [−0.457, − 0.724]) effects. This confirms that DS’ performances were comparable across the two evaluations.

## Discussion

The aim of this study was to determine whether deficits in holistic processing can explain the impairment in recognising faces in an individual with Acquired Prosopagnosia. Furthermore, we wanted to investigate whether these holistic processing impairments (if present) are task-specific (i.e., unique to specific measures of holistic face processing), face-specific and persistent over time. To our knowledge, this is the first longitudinal study to re-examine deficits in Acquired Prosopagnosia using the same holistic processing measures across different time points, with a follow-up assessment four years later. Specifically, this approach allows investigation into whether holistic processing can be improved, relearned, or even deteriorate over time in Acquired Prosopagnosia.

In comparison to the control group, Patient DS was impaired in objective measures of face recognition and face perception, but had comparable non-face object recognition, constituting a case of “pure” Acquired Prosopagnosics (APs)^5,31,53^. Notably, our single-case analyses also revealed that Patient DS was comparable to their demographically-matched controls in the part-whole, composite and Navon’s task, but not in the inversion task, partially replicating previous studies^6,31,32,34,36^. In other words, across the four measures of holistic processing (including faces and non-face objects), demographically-matched NTs showed stronger holistic face processing compared to Patient DS only for the face inversion effect (FIE) (but see below). Additionally, we confirmed that the findings remained consistent even when DS was compared against a larger control sample (20 to 70 years old) after controlling for age, suggesting that a small comparison sample may be less of a limitation. Most importantly, these task-specific holistic deficit(s) persisted at a follow-up evaluation four years later.

In contrast to some previous studies, our findings suggest that not all aspects of holistic processing are necessarily impaired in Acquired Prosopagnosia^5,29,30,33,35^. For instance, Patient DS exhibited a reduced inversion effect but preserved holistic processing in the part-whole and composite effects. This pattern raises the possibility that certain holistic mechanisms may be preserved despite the face recognition difficulties generally observed in APs. In view of this, it is possible that holistic representations of faces are constituted by multiple brain regions in the face processing network, in which APs may have intact and/or spared brain “regions” that are sufficient to form a holistic visual representation of a face^36^. In the case of Patient DS, the cognitive processes underlying the part-whole and composite face effects appeared to be preserved, while those involved in the face inversion effect were impaired.

More interestingly, we found that the pattern of holistic impairment (and preservation) in Patient DS persisted over time, as evidenced by comparable performances during a second evaluation conducted four years later. This suggests that face recognition difficulties linked to holistic processing deficits of faces in Acquired Prosopagnosia are unlikely to be transient but rather *chronic*. Specifically, holistic impairments appear to be stable over time, showing that holistic processing did not improve and/or did not spread to other cognitive mechanisms of holistic processing, even after an extended period. Across two evaluations, our findings tentatively suggests that specific underlying mechanisms of holistic processing are tied to distinct brain regions, and that damage to these regions results in *irreversible* holistic deficits. However, other APs might present a very different pattern of preserved and impaired holistic processing (for example, Patient PS^5^^[,31^^[,34^). In brief, a comprehensive evaluation of holistic processing deficits in AP should include a combination of different holistic processing tasks, measured across distinct time points.

The chronic deficits observed following lesions to regions critical for holistic face processing imply the need for targeted rehabilitative or compensatory strategies to manage face recognition impairments in AP. Recent reviews have emphasised that characterising prosopagnosia subtypes is essential for developing tailored interventions^72^. Consistent with this view, our findings indicate a potential selective impairment in the holistic mechanism indexed by the FIE, but not part-whole or composite effects. While our single-case study showed no improvements after four years, this may not be the case if APs had received targeted training. Notably, Bate et al. ^49^ demonstrated that targeted training increased Patient EM’s FIE magnitude, which was accompanied by a shift towards a more typical scan path when viewing faces and improved performance on the CFPT. This pattern raises the question of whether perceptual training aimed at enhancing holistic processing could selectively improve FIE performance in Patient DS and whether such gains would generalise to everyday face recognition.

Nonetheless, our current study is not without limitations. Our single-case approach may provide valuable insights about the deficits observed in the case of AP, but some caution is needed due to due to floor effects in Patient DS. For example, potential impairments in holistic processing as measured by the part-whole and the face composite tasks in DS could be obscured by chance performances in these tasks. However, given that they have a severe deficit in face recognition, observing chance or close-to-chance performance in APs is not surprising (Case PS ^32^ and Case ST ^30^). An important strength of the control-based regression approach^14,62,63^ adopted here is that it quantifies the predicted performance in the condition of interest based on the control condition, using the normative relationship derived from the NT sample. This approach evaluates the relative difference between conditions, rather than relying solely on raw accuracy scores, and therefore remains informative even when performance is at or near chance. By incorporating both conditions into a single estimate, the approach reduces bias arising from chance-level performance and provides a principled estimate of whether the observed pattern deviates from that expected in NTs. This allows detection of atypical patterns of APs that might otherwise be obscured when interpreting raw scores alone. While the observed temporal stability of task-specific deficits is suggestive of persistent impairment, floor-level raw accuracies remain a potential limitation, and residuals should therefore be interpreted cautiously and conservatively.

Two different problems with our general assessment method must be noted. First, in the second evaluation, we are assuming that DS’ face recognition deficits are still present but the standard AP diagnostic tests (i.e., CFMT, CFPT and CCMT) were not re-administered. However, there is very little to no evidence of spontaneous recovery in AP after several years post-lesion^48^. In addition, DS showed clear face recognition impairments in the holistic processing tasks in the second evaluation as well. Second, the current study did not include measures of low- to mid-level visual processing, which would have complemented the assessment. However, it is unlikely that any of DS’ impairments can be explained at these levels of visual processing, as DS’ performance in the CCMT and the Navon tasks was in the normal range. Nevertheless, future studies may benefit from recruiting a larger sample of APs to clarify whether these holistic face impairments are indeed task-specific and/or heterogeneous, in relation to the different areas of lesion.

Another potential limitation comes from the face inversion task. Compared to the part-whole and composite effects, the FIE has generally been regarded as the most reliable and best predictor of face recognition abilities^12,14,15^. While some studies have suggested that the FIE reflects qualitative differences in face processing, wherein inverting faces disrupts holistic processing and forces observers to rely on featural processing^73^, other recent studies have argued that face recognition in both upright and inverted orientations utilizes similar underlying processes^74–76^. Specifically, inverting faces has been argued to impair broader “information gathering” mechanisms, thereby disrupting both holistic and featural processing of facial information^75–79^. From this perspective, the FIE may be more sensitive than other holistic paradigms, as it captures a broader range of perceptual impairments, including featural processing, a compensatory strategy often reported in AP^48^. In Patient DS, such compensatory featural strategies may be disrupted in the FIE, but not in the part-whole and composite face tasks. Consequently, DS’ task-specific impairments might not reflect impaired holistic processing per se, but rather the higher sensitivity of the face inversion task to subtle deficits in mechanisms other than holistic processing. While our findings are consistent with previous studies showing that the three holistic paradigms rely on distinct cognitive mechanisms^12,14,15^, we cannot entirely rule out the influence of task demands and/or sensitivity of these measures.

## Conclusion

Our results from this experiment suggest that Patient DS, a pure case of Acquired Prosopagnosia, has specific impairment for holistic processing that is captured by the face inversion effects. Interestingly, this pattern of holistic impairment in Patient DS appeared to persist over time, indicating that deficits in holistic processing of faces may be stable and *chronic*. However, given the interpretive constraints associated with floor-level accuracy and the single-case nature of this study, these findings should be interpreted with caution. The sustained deficits also reflect enduring structural or functional anomalies in brain regions critical for holistic processing of faces, suggesting specific rehabilitative strategies or compensatory mechanisms required to manage or improve chronic deficits in APs. Additionally, our results are partially in agreement with previous studies^36^ that suggest holistic representations of faces are distributed across multiple “regions” of the face processing network, allowing holistic processing to be preserved in some cases of Acquired Prosopagnosia. Overall, while our findings align with evidence that holistic face processing is not a unitary mechanism^12,14,15^, the differences in task demands and sensitivity could also potentially account for the present results. Future work using adaptive procedures, easier tasks, or increased trial counts, will be essential to clarify and strengthen the conclusions.

## Methods

### Participants

The single case of AP was referred to as Patient DS (refer below for the patient case history). We also tested 15 White neurotypical adults (7 females), with a mean age of 48.6 years (*SD =* 6 years), that were demographically matched (e.g., ethnicity, age, and nationality) to Patient DS, as controls. A wider group (20 to 70 years old) of White neurotypical adults (*N* = 45), with a mean age of 46.4 years (*SD* = 17 years), were also recruited as controls for the additional analyses.

All participants were recruited through online social media platforms and Testable Minds^80^. In the first evaluation, all participants (Patient DS and NTs) were included in a lucky draw that gifted one out of every five participants an Amazon eGift card valued at £30, as compensation for their time. Patient DS was the only participant recruited for the follow-up (second) evaluation and was provided with the compensation directly. A digital informed consent was obtained from all participants prior to participation. All experimental procedures were approved by the Science and Engineering Research Ethics Committee of the University of Nottingham Malaysia (approval code: BLQZ250920). We confirm that all experiments were performed in accordance with relevant guidelines and regulations.

### Case history

**Fig. 2:**
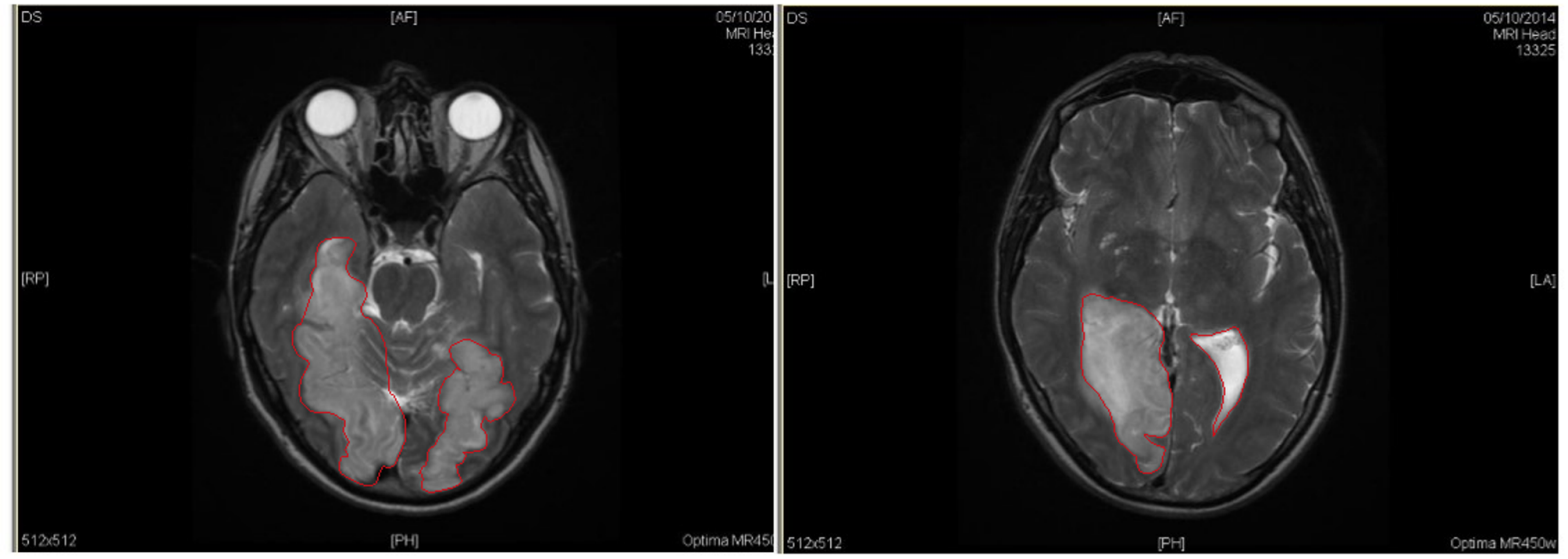
Structural MRI of Patient DS showing the diffuse bilateral brain damage suffered inthe occipital temporal lobes and unilateral damage in the right anterior temporal lobe, following an ischemic stroke (lesion reconstruction in axial view only). *Note.* In axial views, the horizontal orientation is reversed, meaning the left side of the patient’s brain appears on the right side of the image. The diffuse lesion is highlighted approximately within the regions marked in red.

Patient DS is a 55-year-old (at the time of first evaluation) British White male working as an architect, who suffered diffused brain damage in the (right fully and left partially) medial occipitotemporal lobes following an ischemic stroke in his late 40 s (see Fig. 2). Following the brain damage, DS had difficulties recognizing familiar and famous faces when context is removed (e.g., unable to recognize the *Queen* or Winston Churchill when the crown or bowtie were obscured, respectively) and was later diagnosed with AP, acquired topographical agnosia (i.e., difficulties recognizing routes and surrounding landmarks^81^ and aphantasia (i.e., inability to voluntarily create mental images^82,83^, despite normal object recognition.

After the diagnosis, Patient DS received formal cognitive training in using compensatory strategies for face recognition (e.g., semantic, part-based, and mnemonic strategies; for detailed description, see review by Bate & Bennetts^84^, but DS disclosed that these trainings failed to improve his face recognition abilities. DS also confirmed that no specific trainings targeting face recognition and/or holistic processing were attempted during the four-year period between evaluations. When asked to describe his face identification process, Patient DS reported that although he could see faces, he had difficulty looking at multiple facial features simultaneously and retaining facial information in memory. He likened the experience to ‘a magnetic drawing (doodle) board that children play with,’ saying, “I try to draw the face I am looking at in my mind, starting from the left to the right. However, what I’ve drawn disappears as I move along to the right”. In line with his description, DS’ 20-item Prosopagnosia Index (PI20)^85^ score was at the minimum, after removal of items that were not relevant to acquired conditions (e.g., “I have always had a bad memory for faces” and “When I was at school, I struggled to recognize my classmates”). To compensate for his difficulties in identifying individuals, Patient DS attempts identification by focusing on non-facial cues such as body size, hairstyle, biological motion (e.g., gait), smell, and/or voice. At the time of the second evaluation in 2024, it marked the 10-year anniversary of Patient DS’s acquired prosopagnosia diagnosis. DS mentioned that his vision had also been declining since the ischemic stroke, with increasing difficulties perceiving depth and colour. However, these difficulties could also be attributed to age-related cognitive decline.

The deficits described by Patient DS are in line with reviews by Rossion^34^ and Barton et al.^86^. On a behavioural level, Rossion^34^ proposed that previous reports of pure APs (e.g., Patient PS, GG, LR) indicated difficulties in simultaneously integrating multiple regions of a face into a single perceptual representation. Similarly, on an anatomical level, Barton et al.^86^ suggested that four such cases of AP that suffered from lesions in the bilateral occipitotemporal and right anterior temporal lobes presented a combination of apperceptive and amnestic (i.e., associative or non-apperceptive) impairments (refer to Fig. 2). On one hand, apperceptive impairments are shown to be linked with bilateral occipitotemporal damage, wherein APs have deficits in perceiving featural and configural information, as well as facial shapes (e.g., unable to attend multiple aspects of a face). On the other, amnestic impairment was linked to right or bilateral anterior temporal damage and is characterised with normal face matching but degraded face memory as well as impaired imagery (i.e., unable to imagine specific faces)^87^.

### Apparatus

The experimental platform Testable (www.testable.org^80^ was used to conduct this online study, and all tasks were completed on participants’ own computers (laptops or desktops). Testable was a suitable platform for this online study as it standardised the displayed stimuli size across various computer screens by scaling all stimuli according to the number of screen pixels per 1 centimetre (cm). This scaling was calculated when participants adjusted a yellow line on their screen to match the width of a physical debit/credit card. Where appropriate, stimuli were edited using Adobe Photoshop CS6 and Matlab R2019b (Mathworks, Version 9.7.0.1247435) (refer to Stimuli and Procedure).

### Stimuli and procedure

All stimuli and procedure followed in this study were similar to those used in Leong et al.14. Each participant had to complete two different stages over two different days: the “evaluation” stage and the “evaluation” stage (i.e., one stage per day). Participants always completed the “evaluation” stage first, which included non-holistic processing tasks: the CFMT, the CFPT and the CCMT. This was followed by the “experimental” stage, which included the part-whole task, the composite task, the face inversion task and the Navon’s task. The order of the holistic face measures was counterbalanced across all participants. However, the Navon’s task was always completed last as some research has shown that this task could bias responses in subsequent face processing tasks (for discussion, see Estudillo et al.^25^).

Accuracy and reaction time (Navon’s task only) were measured and recorded. To examine the persistence of holistic impairments (if any), Patient DS completed the “experimental” stage again in a second evaluation after four years.

### Evaluation stage

This stage comprised the basic evaluation tasks that assisted in confirming prosopagnosia: the CFMT, CFPT and CCMT. The CFMT was used as a measure of face recognition ability and the CFPT was used to quantify the ability to perceive faces, while the CCMT was used to measure the ability to recognise non-face objects.

### Cambridge face memory test (CFMT)

The stimuli and procedures were the same as the original version of the CFMT^56^. A total of 52 unique White male face identities were used (six target and 46 unique distractor face identities). No identities contained external features, and they all had three different viewpoints (left half-profile, full-frontal and right half-profile). Each face was embedded in the centre of a uniformly black background (195 × 222 pixels (px); 3.9 × 4.44 cm: width × height). The test contained a total of 72 trials, presented in a fixed order. Each trial consisted of three faces (one target and two distractors), where participants were required to press the allocated key to match the target and a learned face. The maximum achievable score on the CFMT is 72, and a score below 42 typically denotes face recognition deficits^14,88–91^.

### Cambridge face perception test (CFPT)

We used the original CFPT from Duchaine et al.^40^, where participants had to sort six test faces (i.e., morphed faces) based on their similarity to a target face in each trial. In this task, eight unique White male face identities were used as target faces. Target faces were always in ¾ profile views, and were embedded on a white background (190 × 190 px; 3.8 × 3.8 cm). To create “test” faces, target faces were morphed (28%, 40%, 64%, 76% and 88%) with one other target face. All test faces were always in their frontal view, and were embedded on a grey background instead. Both test and target faces had external face features covered by a standard black cap and were cropped similarly (e.g., from below the neck).

The test contained a total of 16 *sort* trials, half of the trials were in upright and the other half was presented in an inverted orientation. In each trial, participants were given one minute to reorder the test faces, from the most similar (left) to least similar (right), to a target face. The sum of deviations (i.e., errors) from the correct position for each face (e.g., if a face was placed one position away from its correct location, it was counted as one point) were calculated for upright and inverted items separately. The sum of error scores from the eight upright and the eight inverted items were then averaged to compute the final score (i.e., total errors), with higher scores indicating poorer performance^15,40^.

### Cambridge car memory test (CCMT)

The CCMT used in this study was based on the stimuli and procedures adapted from Dennett et al.^57^. The format of the CCMT is identical to the CFMT (i.e., 72 trials presented in a fixed order), with the exception that the stimuli were computer-generated car images instead of faces. All the computer-generated cars had the same colour, with no identifiable badges or insignias visible. Averaged across all cars and viewpoints, the car images had an approximate size of 465 × 215 px (9.3 × 4.3 cm). The maximum score achievable is 72, with any score below 40 suggesting poor non-face object recognition ability^14,57^.

## Experimental stage

### Face inversion task

A total of 30 face identities in three different viewpoints were used in this task^6,14,15^. All face identities were greyscale male faces, with their hair completely covered by a black seamed cap, and were embedded in a white background (300 × 300 px; 6 × 6 cm).

Similar to the original design^14^, participants were required to match the identity of a target face (i.e., frontal view) to one of three test faces (i.e., ¾ profile view) in each of the experimental trials. All experimental trials began with the presentation of a target face for 400 ms, followed by three simultaneously presented test faces for 2000 ms. A blank screen was then presented until the participant responded. Participants were required to press allocated keys to select which of the test faces matched the target face. Across all trials, each target identity was presented twice – once upright and once inverted. The target identities in one trial were also used as distractor faces in other trials that had a different target identity. The target and test face always had consistent orientation within each trial. The task consisted of a total of 60 randomized trials (30 upright and 30 inverted).

### Part-whole task

The part-whole task used was identical to those in Leong et al.^14^ (see also Estudillo et al.^25^; Wong et al.^92^). Using Adobe Photoshop, the face images were edited to produce novel identities with unique configurations of internal features. Each target face was created by combining sex-specific face templates (consisting of only external features) with internal features (i.e., eyes, nose, and mouth) taken from 12 different individuals (six males, six females). To ensure distinctiveness, no internal features were shared across the six target faces for each gender.

This task consisted of two types of test stimuli, both in greyscale. The first consisted of isolated features (eyes, nose, or mouth only), taken from the original target faces. The second type comprised of modified whole faces (i.e., foils) that were created by replacing one internal feature (eyes, nose, or mouth) of a target face with the corresponding feature from another target face. All isolated features had similar sizes (e.g., eyes: 234 × 80 px; 4.68 × 1.6 cm, nose: 97 × 77 px; 1.94 × 1.54 cm, mouth: 138 × 71 px; 2.76 × 1.42 cm) and was consistent to the features in the original whole faces. All whole faces were embedded in a uniformly grey background (370 × 500 px; 7.4 × 10 cm).

Each trial began with the presentation of a target whole face for 1000 ms, followed by a scrambled face mask (i.e., randomly reordering tiled segments of the face image) shown for 500 ms. Subsequently, two test images were presented side-by-side and remained on the screen until the participant responded. In the “whole” condition, both test images were whole faces. In the “part” condition, they consisted of isolated features (e.g., two eyes) from different target faces. Participants were instructed to identify which of the two test images matched the previously shown target face by pressing one of two allocated keys. The task included 144 randomized trials coming from 2 conditions (whole, part) × 3 features (eyes, nose, mouth). Each condition-feature combination comprised 24 trials, equally distributed between male and female target faces.

### Face composite task

A total of 15 greyscale faces (seven females), all with neutral expressions, were obtained from Retter and Rossion^93^. Composite faces were created by separating the top and bottom halves of faces with a horizontal white gap of three pixels, split at the bridge of the nose (approximately 5% above the nostrils). Five initial composites were created: one consisting of the same identity for both halves, and four combining the top half of one identity with the bottom half of a different identity, matched for gender and face width. These composites were then duplicated to form “misaligned” versions, where the bottom half was shifted 25% to the right. These aligned and misaligned faces served as the “target” stimuli. To reduce the likelihood of participants matching based on low-level visual features^16^, all target composites (227 × 325 px; 4.54 × 6.5 cm) were enlarged by 5% to produce the test stimuli (238 × 350 px; 4.76 × 7 cm).

This procedure adopted the protocols outlined by Susilo et al.^94^ and followed the standard version of the composite task^14,16^. In each trial, the bottom halves of the composite faces always differed between the target and test stimuli, while the top halves were identical in half of the trials (“same” condition) and different in the other half (“different” condition). Participants were instructed to ignore the bottom halves and judge whether the top halves were the same or different. They responded by pressing the one of the two allocated keys. The task comprised 120 randomized trials: 40 same-aligned, 40 same-misaligned, 20 different-aligned, and 20 different-misaligned. In each trial, two composite faces were presented sequentially, each for 400 ms, separated by a 500 ms grey blank screen. Both composite faces in a given trial were always consistent (i.e., either aligned or misaligned).

### Navon’s task

The stimuli and procedure were from Leong et al.^14^. Participants were presented with two types of stimuli (congruent or incongruent), consisting of large letters (‘H’ or ‘S’) composed of smaller letters (‘H’s or ‘S’s). In congruent stimuli, the large and smaller letters were the same (e.g., a large ‘H’ made of small ‘H’s), while in incongruent stimuli, the large and smaller letters differed (e.g., a large ‘H’ made of small ‘S’s). The large letters had a size of 278 × 162 px (5.56 × 3.24 cm), and the small letters measured 37 × 22 px (0.74 × 0.44 cm). All stimuli were white on a 6 × 6 cm black background and centrally presented.

Each participant completed four experimental blocks: two “global” blocks (A) where they were instructed to identify the large (global) letter, and two “local” blocks (B), where they identified the small (local) letters. The blocks were presented in an ABAB order. Across the experiment, participants completed 48 test trials: 24 congruent and 24 incongruent, evenly distributed and randomised within each block. Each trial began with a fixation cross (22 × 22 px; 0.44 × 0.44 cm) displayed for 1000 ms, followed by the stimulus for 180 ms, and then a blank screen that remained until the participant responded.

## Supplementary Information

Below is the link to the electronic supplementary material.

Supplementary Material 1

## Data Availability

The datasets generated during and/or analysed during the current study are available in the Open Science Framework repository, [https://osf.io/kcazn/?view_only=639324528a4e47deb99feda9d506eab5](https:/osf.io/kcazn/?view_only=639324528a4e47deb99feda9d506eab5).
