# Supplementary material for: Persistent task-specific impairment of holistic face processing in acquired prosopagnosia
Source: Sci Rep. 2025 Dec 4;15:43115. doi: 10.1038/s41598-025-28666-3 (PMC12678792; doi:10.1038/s41598-025-28666-3)
Supplement: Supplementary file 1 — Supplementary Material 1 [file 41598_2025_28666_MOESM1_ESM.docx]

**Supplementary Information**

The primary focus in single-case studies is often on establishing dissociations of cognitive function (e.g., differences in performance across tasks). A typical definition or criteria of *classical* dissociation involves a patient being impaired on Task X, normal performance on Task Y, and significantly poorer performance on Task X compared to Task Y ^[1],[2]^. Extending on this, we further compared performances across different holistic measures. For instance, in both evaluations, Patient DS was impaired in one measure (e.g., inversion effect) but comparable to NTs in the second and third measures (e.g., part-whole and composite effects). However, these distinctions are purely based on one comparison being significant and the other(s) not being significant. To fully establish the distinction between tasks, or to confirm whether Patient DS is selectively impaired at some tasks, we ran additional post-hoc analyses to test if there is also a classical dissociation of holistic impairments in Patient DS. To do this, we compared Patient DS’ performances on two holistic tasks using Crawford and Garthwaite’s ^[1]^ revised criteria for classical dissociation.

Our dissociation analyses provide further support to the notion that Patient DS’s impairment was task-specific in the first evaluation (see Table S1). Specifically, the dissociations were generally consistent with our single-case analyses, wherein Patient DS’ performances in the face inversion task showed clear classical dissociations from the performances in the part-whole and composite face tasks. However, although the single-case analyses in the second evaluation four years later showed significant impairment only in the inversion effect, this was not reflected in the dissociation analyses (e.g., dissociation between FIE-CFE and FIE-PWE did not reach significance). One possible explanation is simply that in the second evaluation, Patient DS showed consistent but reduced holistic processing deficits on certain task (i.e., face inversion task) and increased deficits in another task (i.e., composite face task) (see Figure 1). Consequently, in conservative dissociation statistics, these smaller differences across tasks may not be robust enough to achieve statistical significance.

**Table S2.**

*Dissociation analyses of Patient DS and the respective age matched-NTs.*

| **Tasks** | | **Patient DS** | |
| --- | --- | --- | --- |
|  |  | ***M*_1_** | ***M*_2_** |
| **FIE-PWE** | *t* | 2.312* | .899 |
|  | *ZDcc* | -2.531 | -.979 |
|  | 95% CI | [-4.149, -1.148] | [-1.996, -.053] |
| **FIE-CFE** | *t* | 2.369* | .673 |
|  | *ZDcc* | -2.551 | -.723 |
|  | 95% CI | [-3.973, -1.349] | [-1.628, .105] |
| **PWE-CFE** | *t* | .601 | .040 |
|  | *ZDcc* | -.649 | .044 |
|  | 95% CI | [-1.290, -.045] | [-.660, .752] |

*Note*. *M_1_*, first evaluation; *M_2_*, second evaluation; *ZDcc*, Effect size for the difference between suspected DPs and NTs; 95% CI, 95% Bayesian Credible Interval; A significant classical dissociation in the three holistic measures is highlighted in grey; revised standardized difference test: **p* <. 05, ***p* <.01, ****p< .*001 (two-tailed).

Lastly, to further examine if Patient DS have impaired holistic processing only for specific features, we also compared the residuals between DS and age-matched NTs for each feature in the part-whole task (see DeGutis et al. ^[3]^; Leong et al. ^[4]^). Accordingly, our single-case analyses revealed that DS showed a significantly smaller part-whole effect than age-matched NTs for the eyes in the first evaluation, but not in the second evaluation, nor for other facial features across either evaluation.

**Table S1.**

*Single-case analyses of part-whole performance across features in Patient DS and age-matched NTs.*

| **Tasks** | | **Patient DS** | |
| --- | --- | --- | --- |
|  | | ***M*_1_** | ***M*_2_** |
| Eyes | Mean | -.222 | -.080 |
|  | *t* | -3.530** | -1.263 |
|  | *Zcc* | -3.646 | -1.305 |
|  | 95% CI | [-5.065, -2.210] | [-1.989, -.596] |
| Nose | Mean | -.027 | -.089 |
|  | *t* | -.194 | -.633 |
|  | *Zcc* | -.200 | -.654 |
|  | 95% CI | [-0.708, 0.315] | [-1.204, -.084] |
| Mouth | Mean | -.080 | -.194 |
|  | *t* | -.664 | -1.605 |
|  | *Zcc* | - .685 | -1.657 |
|  | 95% CI | [-1.241, -.111] | [-2.436, -.854] |

*Note*. *M_1_*, differences in first evaluation between DS and NTs; *M_2_*, differences in second evaluation between DS and NTs; Residual scores are based on the normative regression lines of NTs’ performances, wherein a significantly different score between Patient DS and the demographically-matched NTs are highlighted in grey; *Z*cc, Effect size for difference between case and controls; 95% CI, 95% confidence interval estimates of the effect size; modified independent *t*-test: **p* <. 05, ***p* <.01, ****p< .*001 (two-tailed).

1. Crawford, J. R., & Garthwaite, P. H. Detecting dissociations in single-case studies: Type I errors, statistical power and the classical versus strong distinction. *Neuropsychologia* **44**, 2249-2258 (2006).
2. Crawford, J. R., & Garthwaite, P. H. Testing for suspected impairments and dissociations in single-case studies in neuropsychology: evaluation of alternatives using monte carlo simulations and revised tests for dissociations. *Neuropsychology* **19**, 318 (2005).
3. DeGutis, J., Cohan, S., Mercado, R. J., Wilmer, J., & Nakayama, K. Holistic processing of the mouth but not the eyes in developmental prosopagnosia. *Cognitive Neuropsychology* **29**, 419-446 (2012).
4. Leong, B. Q. Z., Hussain Ismail, A. M., Wong, H. K., & Estudillo, A. J. The heterogeneity of holistic processing profiles in developmental prosopagnosia: holistic processing is impaired but not absent. *Cognitive Neuropsychology* **41**, 129-147 (2024).
